# Ehlers-Danlos syndrome hypermobility type is associated with rheumatic diseases

**DOI:** 10.1038/srep39636

**Published:** 2017-01-04

**Authors:** Kyla R. Rodgers, Jiang Gui, Mary Beth P. Dinulos, Richard C. Chou

**Affiliations:** 1 Department of Medicine, Geisel School of Medicine at Dartmouth, Lebanon, 03756, NH, USA; 2 Department of Biomedical Data Science, Geisel School of Medicine at Dartmouth, Lebanon, 03756, NH, USA; 3 Department of Community and Family Medicine, Geisel School of Medicine at Dartmouth, Lebanon, 03756, NH, USA; 4 The Dartmouth Institute for Health Policy and Clinical Practice, Lebanon, NH, USA; 5 Department of Pediatrics, Geisel School of Medicine at Dartmouth, Lebanon, 03756, NH, USA; 6 Department of Pathology, Geisel School of Medicine at Dartmouth, Lebanon, 03756, NH, USA; 7 Division of Genetics, Dartmouth-Hitchcock Medical Center, Lebanon, 03756, NH, USA; 8 Division of Rheumatology, Dartmouth-Hitchcock Medical Center, Lebanon, 03756, NH, USA.

## Abstract

We retrospectively analyzed electronic medical records of patients with Ehlers-Danlos Syndrome hypermobility type (HEDS), including demographic information, workup, rheumatological diagnoses in order to determine its association with rheumatological conditions. HEDS Patients were stratified according to level of workup received (no additional work (physical exam only) = NWU, limited workup = LWU, comprehensive workup = CWU)). HEDS patients were predominantly female (21:4, F:M). The percentage of patients with at least one rheumatological condition was significantly correlated with level of workup (NWU, 9.2%; LWU, 33.3%, CWU, 67.1%; p-value < 0.0001). The HLA-B27 antigen was more prevalent (p-value < 2.2 × 10^–8^) in the CWU HEDS patients (23.9%) than in the general population of the United States (6.1%). HEDS with CWU were associated with more rheumatological conditions (i.e. psoriasis, ankylosing spondylitis, rheumatoid arthritis, fibromyalgia) than those with NWU or LWU. In conclusion, HEDS is associated with complicated rheumatological conditions, which are uncovered by comprehensive workup. These conditions require different clinical management strategies than HEDS, and left untreated could contribute to the pain or even physical disability (i.e. joint erosions) in HEDS patients. While the mechanisms underlying these associations are unknown, it is important that all HEDS patients receive adequate workup to ensure a complete clinical understanding for the best care strategy possible.

Ehlers-Danlos Syndrome (EDS) is a heterogeneous group of congenital connective tissue disorders thought to be caused by mutations in genes coding for collagen proteins (i.e. *COL3A, COL5A*) associated with collagen processing (i.e. *PLOD1, ADAMTS2*), or for extracellular matrix components (i.e. *TNXB*)^1^,^2^,^3^. Tissue fragility is the unifying feature among the six subtypes, though the degree to which specific tissues are affected varies tremendously among subtypes^2^. The hypermobility type of EDS (HEDS, also known as EDS Type III) is largely characterized by extreme joint laxity and soft, velvety skin^1^,^2^,^3^,^4^. Joint hypermobility is most pronounced in childhood, and individuals with joint hypermobility are colloquially known as being “double jointed”. Afflicted individuals are often treated as a curiosity and may even find their increased flexibility a boon in athletic endeavors.

Subjects with HEDS first seek medical attention due to persistent back pain and joint pain without an obvious inciting cause. Although they are often dismissed as the ‘benign’ type of EDS, the symptoms of HEDS vary tremendously between affected individuals and impose a significant negative impact on their quality of life^2^,^4^. Due to joint instability, HEDS patients suffer from frequent joint stress and injury, leading to chronic arthralgia, soft-tissue rheumatism (i.e. tendonitis, bursitis, epicondylitis), and myalgia; many also suffer from frequent subluxations and dislocations^1^,^2^,^3^,^4^. Due to the ubiquity of connective tissue, patients are often afflicted with a plethora of non-musculoskeletal symptoms, such as dysautonomia (i.e. positional orthostatic tachycardia, chronic fatigue, etc.), abnormal proprioception, headaches, and gastrointestinal dysmotility, which may lead to nutritional deficits^1^,^2^,^3^,^4^,^5^,^6^. Many of those with HEDS also suffer from mood disorders, including depression and anxiety^2^,^4^,^7^,^8^. To date, the genetic basis of HEDS has not been elucidated and molecular diagnosis is not possible. HEDS patients may see multiple subspecialists without realizing a connection between their joint symptoms and multi-systemic involvement of the disease; they are often dismissed as hypochrondriacs, and report feelings of isolation from the lack of diagnosis^4^,^9^.

Perhaps due to a lack of gravitas surrounding the HEDS diagnosis, management of the disease varies among practitioners, and clinical workup does not often extend beyond the joint and skin examination. This practice may lead to missed diagnoses and failure to properly personalize management strategies for individual patients. In the present study, we aimed to determine whether HEDS patients are predisposed to rheumatological conditions.

## Patients and Methods

The present study was approved by the Institutional Review Board at Dartmouth College in Hanover, New Hampshire, USA and performed in accordance with the approved guidelines and regulations. This study was qualified and approved for exemption from a patient consent requirement by the Institutional Review Board based on the requirements for de-identification. All patients depicted in photographs were consented prior to taking photographs, and every reasonable effort was made to ensure that the photographs were taken in an un-identifiable manner. A list of patients with a diagnosis of HEDS was generated by the data research management at Dartmouth-Hitchcock Medical Center, a large, regional tertiary care center serving populations mainly in the states of New Hampshire, Maine, Massachusetts, upstate New York, and Vermont. We performed a retrospective study of these patients at Dartmouth-Hitchcock Medical Center between July 2009 and June 2015. Once the subjects were identified, detailed chart review of electronic medical records was performed to confirm a diagnosis of HEDS (see next paragraph). Demographic information recorded includes age of diagnosis of HEDS, gender, any rheumatological conditions such as autoimmune diseases, non-autoimmune conditions (i.e. fibromyalgia), structural defects, serological workup, laboratory findings, radiographic studies, or any other diagnostic workup. Subjects were further sorted into the categories of “no rheumatological workup” (i.e. physical examination only), “limited workup” (i.e. only checking one or two laboratory markers, such as anti-nuclear antibodies (ANA) and/or rheumatoid factor (RF)), or “comprehensive workup” (detailed serological markers, radiographic studies, etc.) after a diagnosis of HEDS was made.

### Diagnosis of HEDS

For all patients, a clinical diagnosis of HEDS was made based on the Brighton criteria (Supplementary Note), a refined set of guidelines that takes into consideration hypermobility, as determined by the time-tested Beighton score (Supplementary Note), as well as symptoms of musculoskeletal abnormalities such as arthralgia, soft tissue lesions, etc.^1^. Typical clinical features of a HEDS patient can be seen in Supplementary Figure S1.

### Statistical Analysis

We used the chi-square test to compare the number of patients with rheumatological conditions in each workup group. Additionally, we used chi-square analysis to compare the female to male ratio of HEDS patients diagnosed in either the rheumatology or genetics clinic to that of their respective overall patient populations. Because our patient population was mostly female, we then used a logistical regression model to obtain an adjusted p-value using gender and age as confounders. We calculated the 95% confidence intervals (CI) for the prevalence of each rheumatological condition based on a binomial distribution model. We used sign test to calculate the prevalence of the HLA-B27 antigen in our population versus the general population. In our supplementary studies, we compared the prevalence of each rheumatic condition in our patient population with that of the general population, we used the sign test to calculate the p-value. In cases where the prevalence estimate in our data did not fall into the prevalence estimation range for the general population, we calculated a conservative two-sided p-value by comparing our estimate with the closer, extreme range value using sign test.

## Results

We identified 379 confirmed cases of HEDS in the Dartmouth-Hitchcock Medical Center (Table 1). These patients were predominantly evaluated in the Rheumatology Clinic (53.3%) or Genetics Clinic (43.8%) for their musculoskeletal complaints, i.e. neck pain, back pain, or multiple joint pain. Only 2.9% of patients received a diagnosis of HEDS from a physician outside of these two clinics. Of these confirmed HEDS patients, 320 (84.4%) were female, and 59 (15.6%) were male. It is well-established that many rheumatic diseases, such as rheumatoid arthritis (RA) or systemic lupus erythematosus (SLE), are more common in women. To ensure that the preponderance of female HEDS patients was not due to a sampling bias from the largely female patient population in the Rheumatology clinic, we compared the ratio of female to male HEDS patients with that of the overall patient population in both the Rheumatology and Genetics clinics (Table 2). While the general patient population of the Rheumatology clinic was biased towards females (66.9% female, 33.1% male), the female to male ratio of HEDS patients diagnosed at the Rheumatology Clinic was even more skewed (89.6% female, 10.4% male, p < 0.0001). Though the female to male ratio of the overall patient population of the Genetics clinic is 1:1 (50.1% female, 49.9% male), the HEDS patients diagnosed by this clinic were predominantly female as well (78.3% female, 21.7% male, p < 0.0001). Statistical analysis confirmed that the female predominance among HEDS patients was not a result of sampling bias (Table 2).

**Figure 1:**
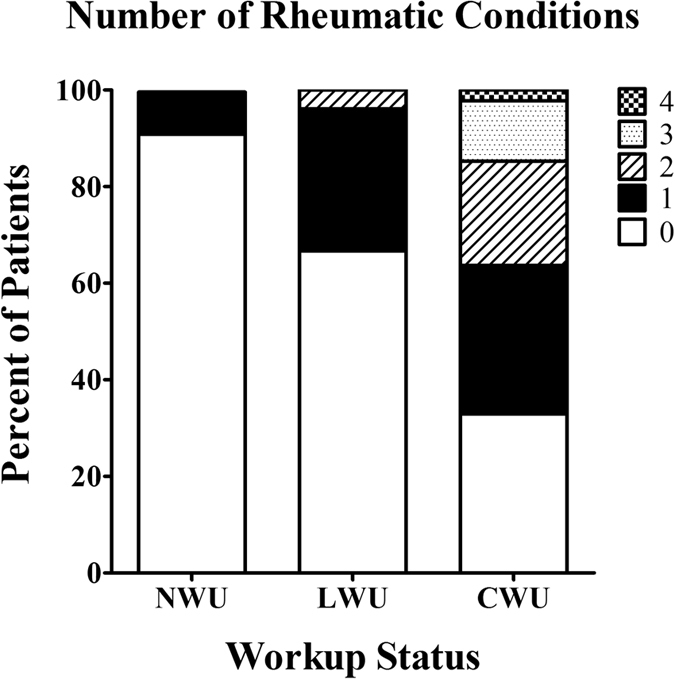
Distribution of number of rheumatological diagnoses by workup status. NWU, no workup; LWU, limited workup; CWU, comprehensive workup.

Next, we classified patients according to the level of workup these HEDS patients received (Table 1). Most patients (240 total) received only a physical examination with no additional workup after a diagnosis of HEDS was made, and were categorized as “no workup” (NWU). Among this group, 21 patients (8.8%) were found to have at least one rheumatological condition in addition to HEDS. A total of 51 patients had limited serological or radiographic studies performed on their spine or joints in addition to the physical examination after a diagnosis of HEDS was made, and were classified as “limited workup” (LWU) patients. Significantly more patients (17, or 33.3%) in the LWU group were found to have at least one rheumatological diagnosis as compared to the NWU patients (adjusted p-value < 0.0001). The remaining 88 patients received a comprehensive workup (CWU), with detailed serological and radiographic studies, after a diagnosis of HEDS was made. Significantly more CWU patients (59, or 67.1%) had at least one rheumatological condition as compared to either the LWU or NWU groups (adjusted p-value < 0.0001). Additionally, patients in the CWU group tended to have more additional diagnoses than patients in either the LWU or NWU groups (Fig. 1); while only a very small percentage of NWU and LWU patients had two additional diagnoses (0.4 and 3.9%, respectively), 21.6% of CWU patients had two additional diagnoses. Furthermore, none of the patients in either the NWU or LWU groups had more than two additional diagnoses, but 12.5% of the CWU presented with three and 2.3% presented with four additional conditions following serological with or without radiographic studies.

Next, we quantified the number of patients who tested positive for serological markers of inflammation or autoimmunity (Table 3), such as ANA, RF, or anti-citrullinated protein antibodies (ACPA); however, it is difficult to determine the significance of these findings because not all patients in the limited workup group were tested for all of these markers. Interestingly, we found that 21 of the patients in the comprehensive workup group (23.9%) were tested positive for HLA-B27 (Table 3). A previous study found that the overall prevalence of the HLA-B27 antigen in the general population of the United States, was 6.1%^10^. Based on this observation, the prevalence of HLA-B27 in our patient population who received comprehensive workup was significantly higher than that of the general population (p-value = 2.2 × 10^−8^).

Next, we catalogued all of the rheumatological conditions seen in our HEDS patients among all the different workup groups. These categories included structural defects (Table 4), non-inflammatory conditions (Table 4), and inflammatory conditions (Table 5). Of the 40 different conditions listed, ten were diagnosed solely on clinical grounds such as structural/physical abnormalities (club feet, developmental delay, occulocutaneous albinism type 1, pectus carinatum, and pectus excavatum) or physical examination (costochondritis, erythromelaliga, fibromyalgia, psoriasis, and Raynaud’s phenomenon); the majority of the conditions require further diagnostic workup, including serological studies (i.e. C3 hypocomplementemia), radiographic studies (i.e. inflammatory arthritis with erosive disease), or skin biopsy (i.e. small fiber sensory neuropathy). The five most common conditions were fibromyalgia (22 cases) and psoriasis (22 cases), followed by ankylosing spondylitis (AS) (11 cases), psoriatic arthritis (PsA) (11 cases), and then RA (9 cases). Interestingly, 27.3% of PsA patients and 33% of RA patients (both seronegative and seropositive) were diagnosed with advanced erosive disease, and without radiographic studies, these patients would have been missed solely based on physical examination. Of all the inflammatory conditions, only psoriasis and Kawasaki disease were diagnosed in the NWU group, as these two conditions require only physical examination for diagnosis. Although a limited workup allowed for six more inflammatory conditions to be diagnosed as compared to the NWU group, there were still ten fewer conditions diagnosed in the LWU than in the CWU group, which may be accounted for by the fact that these conditions require extensive serological and radiographic studies to diagnose.

In an attempt to further quantitate the impact of HEDS on the prevalence of these rheumatological conditions, the prevalence of each rheumatological condition in our patient population was compared to the prevalence of these conditions in the general population, though such comparison does have the potential to produce a sampling bias of these subjects (Supplementary Figure S2). Only the patients in the CWU group were examined because, by definition, the patients in other groups did not receive a sufficient workup to rule out the possibility of rheumatic diseases. We found cases of nine different structural defects (Table 4), three non-inflammatory diseases (Table 4), and nineteen inflammatory diseases (Table 5), for a total of 31 different rheumatalogical conditions among CWU patients. The prevalence for ten of these conditions in the general population cannot be identified (Supplementary Table S2), either because these conditions are too rare or because epidemiological data do not exist. Among the remaining conditions, 15 were significantly more prevalent in our CWU patient population than in the general population (Supplementary Figure S2), including club foot deformity, hereditary angioedema, primary hypogammaglobulemia, fibromyalgia, erythromelalgia, psoriasis, PsA, AS, RA, inflammatory eye disease, autoimmune thyroiditis, SLE, Crohn’s disease, pernicious anemia, and TNF Receptor-Associated Periodic Fever Syndrome (TRAPS).

## Discussion

HEDS has been viewed historically as somewhat benign in the medical field. However, recent studies found that HEDS patients suffer from a constellation of debilitating symptoms: migraine headaches, chronic fatigue, sleep disturbances, functional gastrointestinal disorders, etc. Now, for the first time, we have shown that HEDS is associated with a number of rheumatological conditions, both inflammatory and non-inflammatory. Like many rheumatic diseases, such as RA or SLE, our HEDS patient population was predominantly female (84.4%). To ensure that the preponderance of female HEDS patients in the present study was not due to a sampling bias from the largely female patient population in the Rheumatology clinic, we examined the female to male ratio of HEDS patients in both the Rheumatology and Genetics clinics, and compared this to the gender ratio of the general patient population in each clinic. While Rheumatology patients were predictably predominantly female (66.9% female, 33.1% male), HEDS patients in the Rheumatology clinic were even more heavily affected by a female bias (89.6% female, 10.4% male). Furthermore, the vast majority of HEDS patients in the Genetics clinic were female (78.3% female, 21.7% male), despite a 1:1 female to male ratio in the overall patient population of the Genetics clinic. Although HEDS is considered to be an autosomal dominant trait with complete penetrance, a very similar female to male ratio (89% female, 11% male) has also been reported in an Italian HEDS cohort^5^. A number of explanations, including differences in musculature, sex hormones, and pain perception between men and women have been proposed as potential explanations for the bias; however, as discussed previously, the genetic causes of HEDS remain largely unknown^5^,^11^. Without a definitive genetic basis, any explanation of the strong female bias remains speculative. Further investigation into the genetic mutations that lead to HEDS is required in order to determine whether it could be an X-linked disease.

Additionally, our data show that HEDS patients with back pain, joint pain, and joint laxity were more likely to be diagnosed with at least one rheumatological condition when they received comprehensive serological and radiographic workup for their musculoskeletal complaints in addition to a physical examination (67.1%). When the patients did not receive further workup (NWU) once a diagnosis of HEDS was made, any additional rheumatological diagnosis was made entirely based on physical examination, i.e. psoriasis, fibromyalgia, Raynaud’s phenomenon. Even a limited workup (LWU) revealed significantly more rheumatological conditions among HEDS patients than a simple physical exam (i.e. NWU) did. For example, radiographic studies of HEDS patients further revealed advanced inflammatory arthritis with the presence of joint erosions at the time of workup in the CWU group (27% of patients with PsA, 33.3% of patients with RA; Table 5). Therefore, a more comprehensive workup tended to unveil a more complex clinical picture than a purely physical examination in these patients: nearly 15% of patients who had a comprehensive workup were diagnosed with three or four rheumatological conditions. None in the limited (LWU) or no workup (NWU) groups were found to have more than two rheumatologic diseases for their musculoskeletal complaints. Our findings indicate that a more complex clinical picture may emerge from complaints of back pain, joint pain, and joint laxity following comprehensive serological studies (with or without radiographic studies) than a “straightforward” HEDS diagnosis. Arthralgia is a predominant chief complaint that might lead to a diagnosis of HEDS patients. Similarly, arthralgia is also a major complaint in many rheumatological conditions (i.e. PsA, RA, etc.); yet additional studies are often required to rule out these conditions that cause joint pain. Therefore, our findings strongly support the adoption of comprehensive workup for all HEDS patients as a standard clinical practice.

It is important to note that although the finding of back pain and arthralgia is an overlapping complaint between HEDS and other conditions such as seronegative spondyloarthropathies and fibromyalgia, distinctions were made among them based on well-defined clinical criteria and diagnostic findings to ensure accurate diagnoses during medical record review. For example, in our patient cohort, seronegative spondyloarthropathies were distinguished from HEDS based on established diagnostic criteria. In the case of PsA, psoriasis was noted for all patients in our cohort, and additional imaging studies were used to further characterize the diagnosis (i.e. inflammation with or without joint erosions). Analogously, all AS patients in our cohort tested positive for the HLA-B27 antigen in conjunction with supporting physical examination and radiographic findings. Other seronegative spondyloarthropathy entities also required radiographic/MRI findings of inflammation to confer diagnoses. Thus, although arthralgia is a shared complaint among different disease entities, the use of established clinical criteria and diagnostic findings minimizes ambiguity or uncertainty in each diagnosis. The 2010 American College of Rheumatology diagnostic criteria for fibromyalgia specified that a patient must present with at least 11 out of 18 specified tender points, widespread pain index ≥7, and a score of ≥5 on a symptom severity scale that includes fatigue, waking unrefreshed, cognitive symptoms, and somatic symptoms (i.e. myalgia, numbness/tingling, tinnitus, etc.)^12^. Hypermobile joints are not part of the diagnostic criteria for fibromyalgia, and their presence should lead to consideration of HEDS as a potential additional diagnosis. Thus, a distinction can be made between fibromyalgia-related and HEDS-related complaints (i.e. trigger point tenderness versus Brighton criteria). Nevertheless, any clinical diagnosis is subject to inter-observer variation when more than one clinician is involved in providing care to the patients; therefore, further clinical studies are warranted to verify our findings.

We found that several structural deformities or deficiencies (i.e. club foot, hereditary angioedema, primary hypogammaglobulemia), non-inflammatory diseases (fibromyalgia, erythromelaglia), and autoimmune/inflammatory diseases (psoriasis, PsA, AS, RA, inflammatory eye disease, autoimmune thyroiditis, SLE, Crohn’s disease, pernicious anemia, and TRAPS) were significantly more prevalent in the CWU HEDS population than in the general population of the US. Our analysis of disease prevalence only included CWU patients, as by definition the LWU and NWU patients did not receive sufficient workup to exclude the possibility of additional rheumatological diagnoses (Supplementary Figure S2). However, our statistical analysis does have the potential to produce a sampling bias.

While we confirmed our hypothesis that a number of rheumatological conditions are associated with HEDS, the mechanisms underlying these increased risks are currently unknown, as very little is known about the molecular cause of HEDS. Furthermore, each of these rheumatological conditions has a different underlying disease mechanism; it is currently unknown how one genetic disorder as seen in HEDS could increase the risks of multiple rheumatological conditions. Future studies investigating the genetic and biochemical underpinnings of this congenital disorder are needed to elucidate possible links between HEDS and rheumatological conditions.

## Additional Information

**How to cite this article**: Rodgers, K. R. *et al*. Ehlers-Danlos syndrome hypermobility type is associated with rheumatic diseases. *Sci. Rep.* **7**, 39636; doi: 10.1038/srep39636 (2017).

**Publisher's note:** Springer Nature remains neutral with regard to jurisdictional claims in published maps and institutional affiliations.

## Supplementary Material

Supplementary Information

## Figures and Tables

**Table 1:** Demographics of HEDS patients.

		Number of pts	Average Age at Diagnosis	Age Range at Diagnosis	# w/≥1 Rheum Diagnosis	P-value	
**NWU**	F	195 [81.3%]	29.4	1–74	20 [10.3%]	**Unadjusted**	**Adjusted**
	M	45 [18.7%]	15.4	1–57	1 [2.2%]		
	Total	240	—	—	**21 [8.8%]**		
**LWU**	F	39 [76.5%]	29.5	4–65	12 [30.8%]	<0.0001****	<0.0001****
	M	12 [23.5%]	13.7	4–38	5 [41.7%]		
	Total	51	—	—	**17 [33.3%]**		
**CWU**	F	86 [97.7%]	39.1	11–71	57 [66.3%]	<0.0001****	<0.0001****
	M	2 [2.3%]	23.5	23–24	2 [100.0%]		
	Total	88	—	—	**59 [67.1%]**		

**Table 2:** Gender ratio of HEDS patients compared to general patient population by subspecialty.

	Population	Female	Male	P-value
**Rheumatology**	Clinic	66.9% [9903]	33.1% [4901]	<0.0001****
	HEDS	89.6% [181]	10.4% [21]	
**Genetics**	Clinic	50.1% [1118]	49.9% [1114]	<0.0001****
	HEDS	78.3% [130]	21.7% [36]	

**Table 3:** Laboratory findings in HEDS patients.

		Number of pts	ANA+	RF+	ACPA+	HLA B27+
**LWU**	F	39	6 [15.4%]	1 [2.6%]	1 [2.6%]	—
	M	12	4 [33.3%]	0	0	—
	Total	51	10 [19.6%]	1 [2.0%]	1 [2.0%]	—
**CWU**	F	86	9 [10.5%]	7 [8.1%]	3 [3.5%]	20 [23.3%]
	M	2	0	0	0	1 [50.0%]
	Total	88	9 [10.2%]	7 [8.0%]	3 [3.4%]	21 [23.9%]

**Table 4:** Structural defects and non-inflammatory conditions in HEDS patients.

Diagnosis	NWU	LWU	CWU	
**Structural defects/other**	Scoliosis	1 [0.4%]	1 [2.0%]	5 [5.7%]
	C3 hypocomplementemia	0	1 [2.0%]	1 [1.1%]
	Pectus excavatum	1 [0.4%]	0	1 [1.1%]
	Club feet	0	0	1 [1.1%]
	Developmental delay	1 [0.4%]	0	0
	Early onset generalized osteoarthritis	0	0	1 [1.1%]
	Hereditary angioedema	0	0	1 [1.1%]
	Oculocutaneous albinism type 1	1 [0.4%]	0	0
	Pectus carinatum	1 [0.4%]	0	0
	Primary hypogammaglobulemia	0	0	1 [1.1%]
	Scheuermann’s disease	0	0	1 [1.1%]
	Spina bifida occulta	0	0	1 [1.1%]
**Non-inflammatory diseases**	Fibromyalgia	10 [4.2%]	4 [7.8%]	8 [9.1%]
	Small fiber sensory neuropathy	0	0	8 [9.1%]
	Raynaud’s phenomenon	1 [0.4%]	1 [2.0%]	4 [4.6%]
	Erythromelalgia	0	0	1 [1.1%]
	von Willebrand disease	0	1 [2.0%]	0

**Table 5:** Inflammatory diseases in HEDS patients.

Diagnosis		NWU	LWU	CWU
Psoriasis		6 [2.5%]	1 [2.0%]	15 [17.1%]
Ankylosing spondylitis		0	2 [3.9%]	9 [10.2%]
Psoriatic arthritis	w/o erosions	0	0	8 [9.1%]
	w/erosions	0	0	3 [3.4%]
Seronegative rheumatoid arthritis	w/o erosions	0	2 [3.9%]	2 [2.3%]
	w/erosions	0	0	1 [1.1%]
Seropositive rheumatoid arthritis	w/o erosions	0	1 [2.0%]	2 [2.3%]
	w/erosions	0	0	1 [1.1%]
Inflammatory eye disease		0	0	4 [4.6%]
Systemic lupus erythematosus		0	1 [2.0%]	2 [2.3%]
Autoimmune thyroiditis		0	0	2 [2.3%]
Diffuse enthesopathy		0	0	2 [2.3%]
Juvenile inflammatory arthritis		0	2 [3.9%]	0
Undifferentiated spondyloarthropathy		0	0	2 [2.3%]
Celiac disease		0	0	1 [1.1%]
Costochondritis		0	1 [2.0%]	0
Crohn’s disease		0	0	1 [1.1%]
Erythema nodosum		0	0	1 [1.1%]
Kawasaki disease		1 [0.4%]	0	0
Mixed connective tissue disease		0	0	1 [1.1%]
Pernicious anemia		0	0	1 [1.1%]
Polymyalgia rheumatica		0	1 [2.0%]	0
Sacroiliitis		0	0	1 [1.1%]
Sjogren’s disease		0	0	1 [1.1%]
Seronegative tenosynovitis		0	0	1 [1.1%]
TRAPS		0	0	1 [1.1%]
