# Supplementary material for: Ehlers-Danlos syndrome hypermobility type is associated with rheumatic diseases
Source: Sci Rep. 2017 Jan 4;7:39636. doi: 10.1038/srep39636 (PMC5209734; doi:10.1038/srep39636)
Supplement: Supplementary Information [file srep39636-s1.pdf]

Supplementary Information
Scientific Reports

Ehlers-Danlos syndrome hypermobility type is associated with rheumatic diseases

Kyla R Rodgers, BS[1], Jiang Gui, PhD[2,3,6] , Mary Beth P. Dinulos, MD[4,5,7], and *Richard C. Chou, MD, PhD[1,8]

Departments of Medicine[1], Biomedical Data Science[2], Community and Family Medicine[3], Pediatrics[4], and Pathology[5] Geisel School of Medicine at Dartmouth; The Dartmouth Institute for Health Policy and Clinical Practice[6]; Divisions of Genetics[7] and Rheumatology[8], Dartmouth-Hitchcock Medical Center.

*Address reprint requests and correspondence to:

Richard C. Chou, MD PhD
Department of Medicine
Geisel School of Medicine at Dartmouth
One Medical Center Drive
Lebanon, NH 03756

Email: Richard.c.chou@dartmouth.edu.

Phone: 603-650-8622

Fax: 603-650-4961

Supplemental Note. Brighton criteria for HEDS diagnosis

Requirements for Diagnosis:
Any *<u>one</u>* *of the following:*

- Two major criteria
- One major plus two minor criteria
- Four minor criteria
- Two minor criteria and a family history (positive diagnosis of a first degree relative)

Major Criteria:

- Beighton score ≥4 (9 possible points, scale below)
  - Passive dorsiflexion of the fifth metacarpophalangeal joint to ≥90° (1 point for each side)
  - Passive apposition of the thumb to the flexor side of the forearm while shoulder is flexed 90°, elbow is extended and hand is pronated (1 point for each side)
  - Passive hyperextention of the elbow ≥10° (1 point for each elbow)
  - Passive hyperextention of the knee ≥10° (1 point for each knee)
  - Forward flexion of the trunk, with the knees straight, so that the hand palms rest easily on the floor (1 point)
- Arthralgia in 4 or more joints (>3 months)

Minor Criteria:

- Beighton score of 1, 2, or 3 (see scoring system above)
- Arthralgia in 1-3 joints (>3 months) or back pain or spondylosis/spondylysis/spondylolisthesis (>3 months)
- Dislocation or sublaxation in more than one joint, or in one joint on more than one occasion
- At least 3 soft tissue lesions (i.e. epicondylitis, tenosynovitis, bursitis)
- “Marfanoid” body-type
  - Tall, slim stature
  - Arm span greater than height (>1.03 ratio)
  - Lower segment ratio less than 0.89
  - Long, thin fingers/arachnodactyly
  - High, arched palate
  - Pes planus deformity (flat feet)
- Abnormal skin: hyperextensibility, thin skin, atypical wound healing
- Ocular signs: drooping eyelids, myopia, antimongoloid slant
- Cardiovascular signs: varicose veins, hernia, mitral valve prolapse
- Uterine or rectal prolapse

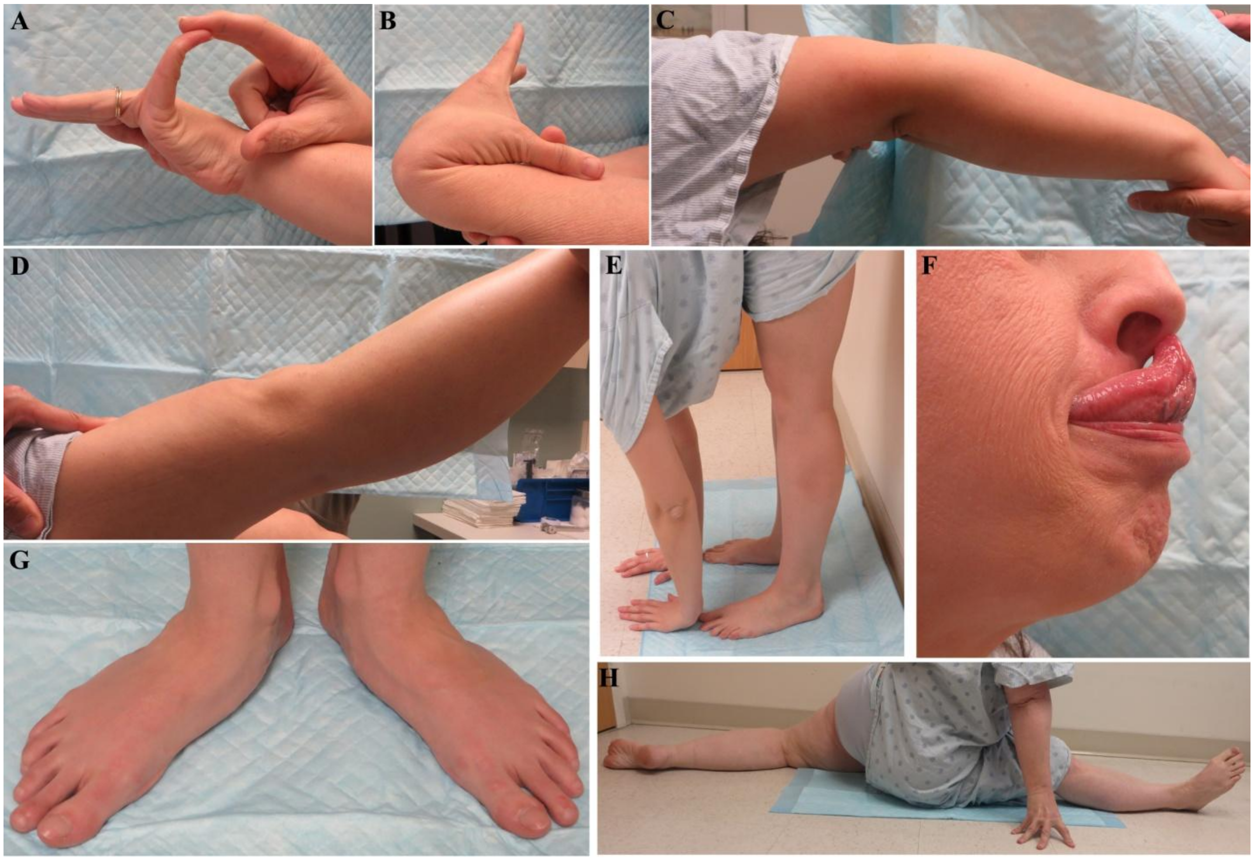

Supplementary Figure S1. Examples of positive Beighton score (A-E) and typical clinical feature of HEDS (F-H). A) Passive dorsiflexion of the fifth metacarpophalangeal joint to ≥90°; B) Passive apposition of the thumb to the flexor side of the forearm; C) Passive hyperextention of the elbow ≥10°; D) Passive hyperextention of the knee ≥10°; E) Forward flexion of the trunk, with the knees straight, so that the hand palms rest easily on the floor; F) Positive Gorlin sign; G) Pes planus deformity of both feet ; H) Hyperflexibility, demonstrated as the ability to do a front split.

Supplementary Table S1. Prevalence of rheumatic conditions with unknown epidemiology among HEDS patients

	**HEDS patients (%)**	**95% Confidence Interval (%)**	**General population (%)**
C3 hypocomplementemia	1.1	[0.3, 6.2]	unknown
diffuse enthesopathy	2.3	[0.3, 8.0]	unknown
early onset generalized osteoarthritis	1.1	[0.3, 6.2]	unknown
erythema nodosum	1.1	[0.3, 6.2]	unknown
Monoclonal gammopathy of unknown significance	1.1	[0.3, 6.2]	unknown
mixed connective tissue disorder	1.1	[0.3, 6.2]	rare, unknown
sacroiliitis	1.1	[0.3, 6.2]	unknown
seronegative tenosynovitis	1.1	[0.3, 6.2]	unknown
small fiber sensory neuropathy	9.1	[3.3, 15.7]	rare, unknown
undifferentiated seronegative spondylarthropathy	2.3	[0.3, 8.0]	unknown

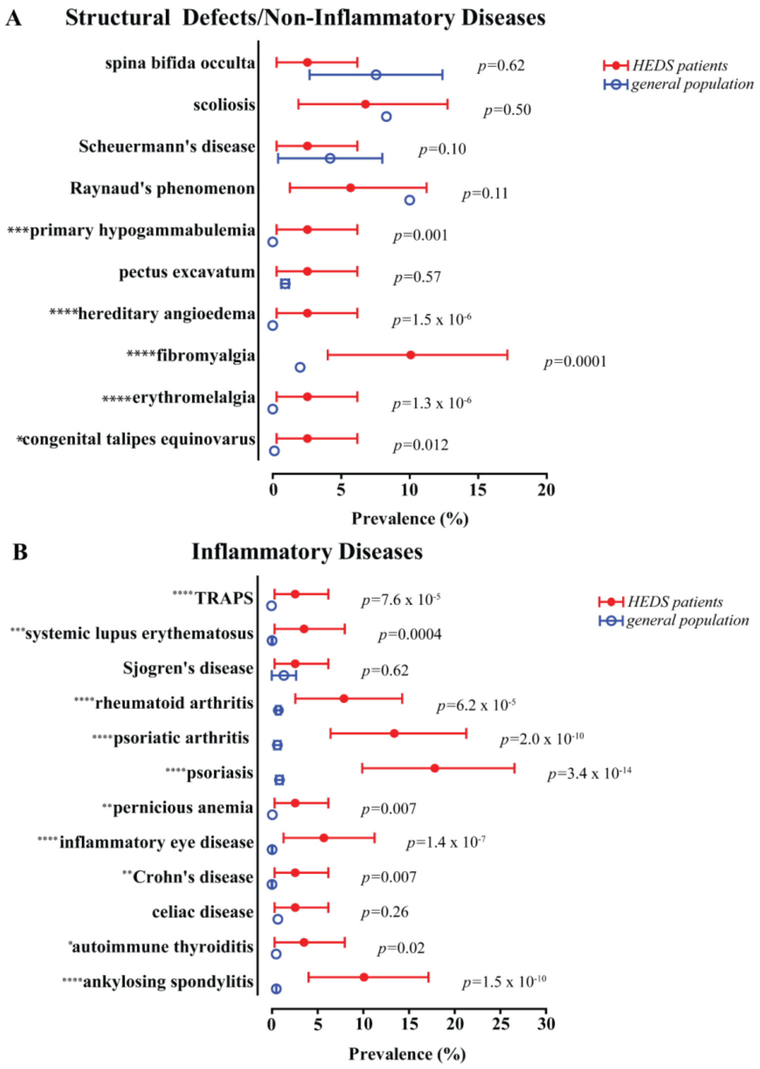

Supplementary Figure S2. Prevalence of structural defects/non-inflammatory diseases (A) and inflammatory diseases (B) among HEDS patients compared to the general population. The bars (red) represent the 95% confidence interval for the prevalence of these diseases among HEDS patients. Blue bars represent the range of estimated prevalence among the general population (see Supplemental Table 3 for references). Abbreviations: TRAPS, tumor necrosis factor-receptor associated periodic syndrome

Supplementary Table S3. Prevalence of rheumatic diseases in the general public

	Prevalence in general population	Source
ankylosing spondylitis	0.52-0.55	Reveille and Weisman, 2013[1]
autoimmune thyroiditis	0.52	McGrogan et al, 2008[2]
celiac disease	0.71	Rubio-Tapia et al, 2012[3]
congenital talipes equinovarus	0.13	Parker et al, 2009[4]
Crohn's disease	0.01-0.1	Loftus et al, 1998[5]
erythromelalgia	0.0013	Reed and Davis, 2009[6]
fibromyalgia	2.0	Wolfe et al, 1995[7]
hereditary angioedema	0.002	Lumry, 2013[8]
inflammatory eye disease	0.038-0.115	Denniston et al, 2013[9]
pectus excavatum	0.628-1.2	de Oliveira et al, 2014[10]
pernicious anemia	0.1	Andres and Serraj, 2012[11]
primary hypogammaglobulemia	0.002-0.004	Engelhardt, 2010[12]
psoriasis	0.51-1.23	Takeshita et al, 2015[13]
psoriatic arthritis	0.3-1.0	Gladman et al, 2005[14]
Raynaud's phenomenon	10.0	Hotchberg et al (editors), 2008[15]
rheumatoid arthritis	0.5-1.0	Helmick et al, 2008[16]
Scheuermann's disease	0.4-8.0	Lowe, 1999[17]
scoliosis	8.3	Carter et al, 1987[18]
Sjogren's disease	0.03-2.7	Patel and Shahane, 2014[19]
spina bifida occulta	2.7-12.4	Eubanks et al, 2009[20]; Parker et al 2010[21]
systemic lupus erythematosus	0.015-0.13	Hotchberg et al (editors), 2008[15]
TRAPS	0.001	Lachmann et al, 2014[22]

REFERENCES:

1. Reveille JD, Hirsch R, Dillon CF, Carroll MD, Weisman MH. The prevalence of HLA-B27 in the US: data from the US National Health and Nutrition Examination Survey, 2009. *Arthritis and rheumatism.* May 2012;64(5):1407-1411.
2. McGrogan A, Seaman HE, Wright JW, de Vries CS. The incidence of autoimmune thyroid disease: a systematic review of the literature. *Clinical endocrinology.* Nov 2008;69(5):687-696.
3. Rubio-Tapia A, Ludvigsson JF, Brantner TL, Murray JA, Everhart JE. The prevalence of celiac disease in the United States. *The American journal of gastroenterology.* Oct 2012;107(10):1538-1544; quiz 1537, 1545.
4. Parker SE, Mai CT, Strickland MJ, et al. Multistate study of the epidemiology of clubfoot. *Birth defects research. Part A, Clinical and molecular teratology.* Nov 2009;85(11):897-904.
5. Loftus EV, Jr., Silverstein MD, Sandborn WJ, Tremaine WJ, Harmsen WS, Zinsmeister AR. Crohn's disease in Olmsted County, Minnesota, 1940-1993: incidence, prevalence, and survival. *Gastroenterology.* Jun 1998;114(6):1161-1168.
6. Reed KB, Davis MD. Incidence of erythromelalgia: a population-based study in Olmsted County, Minnesota. *Journal of the European Academy of Dermatology and Venereology : JEADV.* Jan 2009;23(1):13-15.
7. Wolfe F, Ross K, Anderson J, Russell IJ, Hebert L. The prevalence and characteristics of fibromyalgia in the general population. *Arthritis and rheumatism.* Jan 1995;38(1):19-28.
8. Lumry WR. Overview of epidemiology, pathophysiology, and disease progression in hereditary angioedema. *The American journal of managed care.* Jun 2013;19(7 Suppl):s103-110.
9. Denniston AK, Dick AD. Systemic therapies for inflammatory eye disease: past, present and future. *BMC ophthalmology.* 2013;13:18.
10. de Oliveira Carvalho PE, da Silva MV, Rodrigues OR, Cataneo AJ. Surgical interventions for treating pectus excavatum. *The Cochrane database of systematic reviews.* 2014;10:CD008889.
11. Andres E, Serraj K. Optimal management of pernicious anemia. *Journal of blood medicine.* 2012;3:97-103.
12. Engelhardt KG, B; Herholz, P. Common variable immunodeficiency. http://www.orpha.net/consor/cgi-bin/Disease_Search.php?lng=EN&data_id=3469&Disease_Disease_Search_diseaseType=ORPHA&Disease_Disease_Search_diseaseGroup=1572&Disease(s)/group%20of%20diseases=Primary-hypogammaglobulinemia&title=Primary-hypogammaglobulinemia. Accessed February 2016.
13. Takeshita J, Gelfand JM, Li P, et al. Psoriasis in the US Medicare Population: Prevalence, Treatment, and Factors Associated with Biologic Use. *The Journal of investigative dermatology.* Dec 2015;135(12):2955-2963.
14. Gladman DD, Antoni C, Mease P, Clegg DO, Nash P. Psoriatic arthritis: epidemiology, clinical features, course, and outcome. *Annals of the rheumatic diseases.* Mar 2005;64 Suppl 2:ii14-17.
15. Hochberg MS, AJ; Smolen, JS; Weinblatt, ME; Weisman, MH, ed *Rheumatology.* 4 ed2008.
16. Helmick CG, Felson DT, Lawrence RC, et al. Estimates of the prevalence of arthritis and other rheumatic conditions in the United States. Part I. *Arthritis and rheumatism.* Jan 2008;58(1):15-25.
17. Lowe TG. Scheuermann's disease. *The Orthopedic clinics of North America.* Jul 1999;30(3):475-487, ix.
18. Carter OD, Haynes SG. Prevalence rates for scoliosis in US adults: results from the first National Health and Nutrition Examination Survey. *International journal of epidemiology.* Dec 1987;16(4):537-544.
19. Patel R, Shahane A. The epidemiology of Sjogren's syndrome. *Clinical epidemiology.* 2014;6:247-255.

20. Eubanks JD, Cheruvu VK. Prevalence of sacral spina bifida occulta and its relationship to age, sex, race, and the sacral table angle: an anatomic, osteologic study of three thousand one hundred specimens. *Spine.* Jul 1 2009;34(15):1539-1543.

21. Parker SE, Mai CT, Canfield MA, et al. Updated National Birth Prevalence estimates for selected birth defects in the United States, 2004-2006. *Birth defects research. Part A, Clinical and molecular teratology.* Dec 2010;88(12):1008-1016.

22. Lachmann HJ, Papa R, Gerhold K, et al. The phenotype of TNF receptor-associated autoinflammatory syndrome (TRAPS) at presentation: a series of 158 cases from the Eurofever/EUROTRAPS international registry. *Annals of the rheumatic diseases.* Dec 2014;73(12):2160-2167.
